# Effect of larval handling on black soldier fly life history traits and bioconversion efficiency

**DOI:** 10.3389/fvets.2024.1330342

**Published:** 2024-01-15

**Authors:** Zaira Loiotine, Laura Gasco, Ilaria Biasato, Andrea Resconi, Sara Bellezza Oddon

**Affiliations:** Department of Agricultural, Forest and Food Sciences, University of Turin, Grugliasco, TO, Italy

**Keywords:** *Hermetia illucens*, handling, growth performance, bioconversion efficiency, adult stage

## Abstract

**Introduction:**

The black soldier fly is considered the most promising insect species for mass production; however, information on the effects of handling, which is unavoidable during experimental trials and rearing practices, is still limited.

**Materials and methods:**

To address this gap, three different manipulation intensities were tested on 100 6-day-old larvae per replica (6 replicates/treatments) fed on Gainesville diet: (1) hard-handled (HH), larvae underwent continuous manipulation until the end of larval stage, (2) soft-handled (SH), larvae were manipulated after the appearance of the first prepupa, (3) no-handled (NH), larvae remained untouched. Every 4 days from the beginning to the end of the larval stage, the manipulations lasted 30 min and occurred under laboratory conditions (20°C). During the sampling operations, at least 30 larvae were randomly extracted, washed, dried, and weight-mimicked. At the end of larval stage, all the boxes remained untouched until the adult fly stage, and the emergency rate and sex ratio were evaluated on dead flies. Data were statistically analyzed using IBM SPSS V20.0.0 software and the considered significance level was *p* < 0.05.

**Results:**

The larval stage lasted 8.2 days for both HH and SH (*p* > 0.05). Despite the HH larvae being the most manipulated, no difference was also observed in final weight (HH, 160 mg; SH, 150 mg; *p* > 0.05) and survival rate (HH, 96.2%; SH, 94.5%; *p* > 0.05). The manipulation did not influence the bioconversion capacity of the larvae (bioconversion efficiency corrected for the residue: HH, 14.3%; SH, 12.91%; reduction rate: HH, 58.4%; SH, 55.9%; waste reduction index: HH, 7.28%/day; SH, 7.25%/day; *p* > 0.05). Finally, the development time from larva to fly (about 20.7; *p* > 0.05), the emergency rate (NH: 92.8%; SH: 89.5%; HH: 82.7%) and sex ratio (~1.2% to male flies) were not affected by the handling (*p* > 0.05).

**Discussion:**

In conclusion, the handling procedures used in the current study did not influence the life history traits of the black soldier fly. However, further studies are needed to evaluate if different experimental protocols on various scales, the colony strain or other handling procedures may suggest a different scenario or confirm the results.

## Introduction

The words “*handling* – or – *manipulation*” represent an action that could be able to inflict stress on a biological system (1). In literature, there is little evidence about the effects of handling on the black soldier fly (BSF) development, bioconversion efficiency and survival rate. A search in the SCOPUS database containing the keywords (“black soldier fly” OR “*Hermetia illucens*”) AND (handl* OR manipulat*) in the title, abstract and keywords of the papers yielded a total of 69 studies. Among these studies, the main links between the words “*handling* – or – *manipulation*” and “black soldier fly”—or—“*Hermetia illucens*” are based on handling of waste, diets, manure or bacteria (2–4).

Handling stress has been cited as one of the factors that can negatively affect animal welfare (5). There are several studies that have evaluated the effect of handling on animals with temperament (6). In this regard, a recent literature review by Mota-Rojas et al. (7) highlighted that handling is often able to compromise the production and reproductive processes of farms, especially when it is continued or overdone (i.e., *“hard handling”*). For instance, in dairy cow farms, Arias and Špinka (8) found that cows showed lower milk production, low percent protein and fat, and a positive correlation with cortisol concentration in milk, as well as higher veterinary costs, when experiencing hard handling. In pigs, hard handling reduced testicular size and delayed coordinated mating response, as well as pregnancy rates in sows (9). Furthermore, in poultry, the handling procedures were cited as both responsible for injury and reduced meat quality during catching operations (10), as well as capable of increasing plasma corticosterone levels as an indicator of stress (11).

As suggested by Barrett et al. (5), for insects—being animals (12)—it is reasonable to assume that, in line with what happens with mammals, they may also be affected by reduced welfare with stressful events such as handling. Based on this assumption, in the “fight-or-flight” reaction due to stressors such as handling, while in vertebrates, the adrenal medulla produces a hormonal cascade that leads to the secretion of catecholamines, including norepinephrine and adrenaline (13), in insects as invertebrates, the functions of catecholamines are fulfilled by a major biogenic amine, octopamine, along with dopamine and serotonin (14). Manipulation influence was investigated in the past in insects, locusts (*Locusta schistocerca americana gregaria*), cockroaches (*Periplaneta americana*) and honeybees (*Apis melrifera L.*) (13, 15). These studies measured octopamine levels in species following different types of stress and showed that independent of the type of stress (mechanical, thermal or chemical) (13) and (physical) (15), there was an increased levels of the biogenic amine octopamine. Beyond biogenic amine production, there is little evidence of the effect of manipulation on growth performance in BSF larvae. However, several authors have speculated on the effect of handling BSF larvae in their studies. For example, Nguyen et al. (16, 17) divided the number of replicates between manipulated and non-manipulated larvae. Khaekratoke et al. (18) increased the number of replicates to reduce the effects of manipulation. Miranda et al., (19) established a minimum number of larvae sampled. Meneguz et al. (20) chose not to handle larvae until a defined percentage of prepupae was reached. Bosch et al. (21) also cited the manipulation process in their scientific review and suggested a negative effect of manipulation on survival, which could affect the bioconversion efficiency of the larvae (22). However, there is a dearth of studies looking at the effects of manipulation on the growth performance of BSF larvae.

Considering the lack of insect welfare regulation (23), as well as the increased growth that they are receiving worldwide (24), it is important to develop safe and standardized breeding practices that also take into account the required—and almost unavoidable—handling processes. For example, during the industrial rearing process, it may be necessary to manipulate the larvae to change the feeding substrate, monitor their development, and collect or “pre-process” the larvae (5). Similarly, the type of experiment—small- or large-scale—could also be affected by the manipulation process. Due to the space constraints and the larvae production needed, the small-scale trials currently represent the majority of the studies since they can be performed in laboratory conditions (2). In small-scale trials (e.g., 100 larvae), the percentage of larvae that are manipulated on the total biomass (individual collection, washing, drying and weighting procedures) is higher than in large-scale ones (e.g., 10,000 larvae), where growth parameters are obtained by estimation. Therefore, when assessing the “stress response” to the handling procedures, it is important to consider that it may change depending on the experimental plan, sampling method, frequency and type of handling (e.g., *“hard handling”* or *“gentle* or *soft handling”*) (25).

To the authors’ knowledge, the present study is the first one that aims to evaluate the effect of manipulation on BSF larval growth, bioconversion efficiency and adult emergence. For this reason, the results can contribute to improve research protocols and clarify arguments in the article discussions. As previously reported, since most of the trials are generally conducted in a small-scale, this kind of experimental set-up was considered.

## Materials and methods

### Colony status, maintenance and infrastructures

The BSF colony is situated at the Experimental Facility of the Department of Agricultural, Forest and Food Sciences (University of Turin) and it is housed into three climatic chambers (MONTI & C.—Tecnologie del Freddo S.r.l.; Potenza, Italy) designated for the reproduction (T°: 30° ± 0.5°C, relative humidity: 75 ± 5%, light:dark = 12:12), and the larval stage and the experimental trials (T°: 28 ± 0.5°C; relative humidity: 70 ± 5%, light:dark = 0:24). All the life stages are subjected to periodically hygiene and health checks by the national reference institution. The larvae are fed on Gainesville diet and the primary management operations to maintain the life cycle include eggs collection, neonatal care and larvae estimation at 6-day-old, following the procedures outlined by Deruytter et al. (26). Additionally, the sieving procedure is implemented at the end of the larval stage (16 day old).

### Experimental diet

The Gainesville diet (27), composed by wheat bran, alfalfa and corn (30.1% dry matter, 14.9% crude protein, 2.1% ether extract; 6% ash on dry matter) served as substrate in a single-batch feeding system (1.8 g/larva, KERN, GAB-N, d, 0.1 g). The Gainesville diet was prepared using warm tap water and then 180 grams were placed inside each trial box with perforated lid (15 cm × 15 cm × 5 cm). Subsequently, the boxes were transferred to the climatic chamber to acclimatize the substrates, ensuring that the core temperature reached 23 ± 0.5°C. This pre-heated step aimed to prevent thermal shock resulting from a rapid temperature change, which could otherwise reduce the metabolism of the larvae during their inoculation.

### Experimental set-up

**Figure 1:**
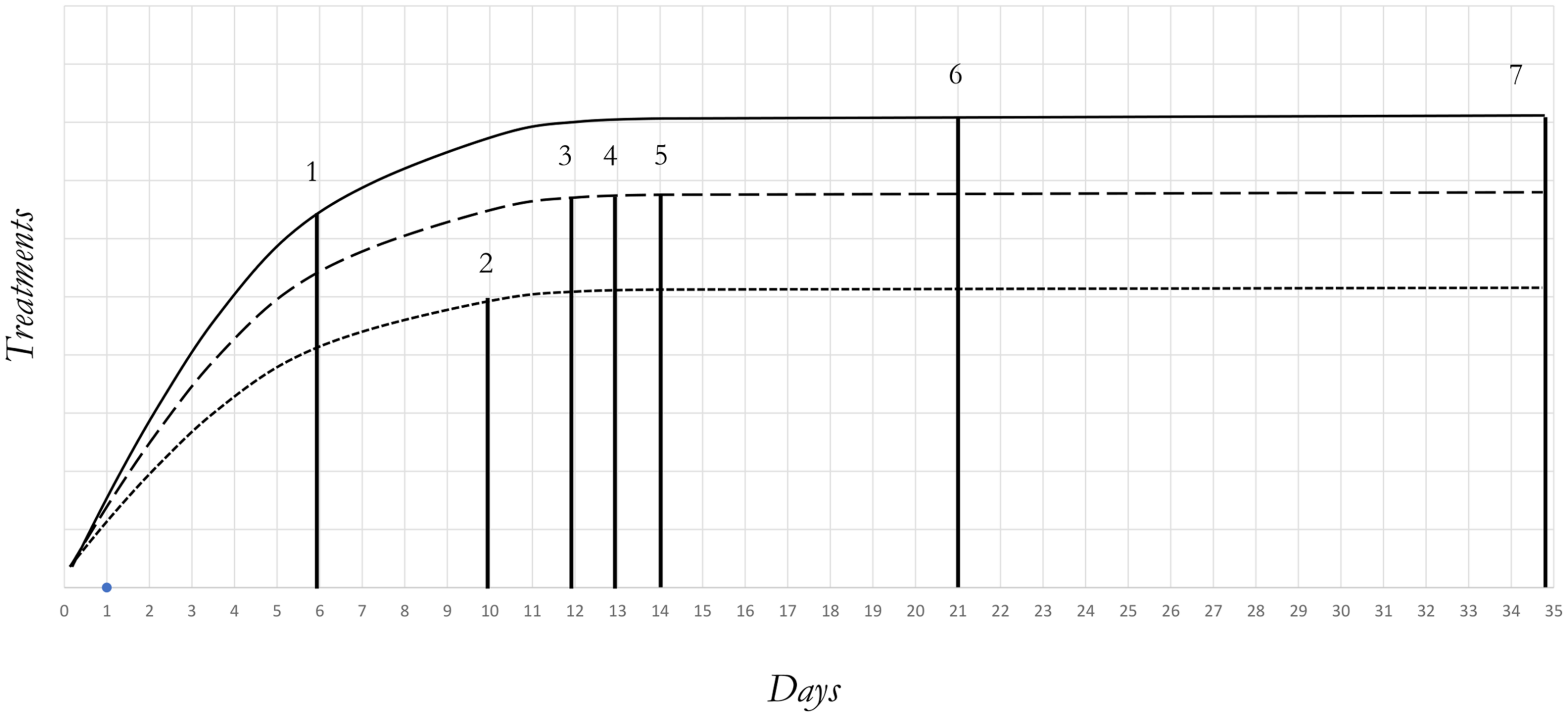
Illustration of handling and time procedures for NH, SH and HH treatments during the trial. Legend: NH (no handling); SH (soft handling); HH (hard handling); DO (days old). NH (—); SH (− − –); HH (−----); 1 (initial sampling); 2 (second sampling); 3 (first prepupae search); 4 (5% prepupae search); 5 (final sampling—5% prepupae achieved); 6 (first fly emerged); 7 (dead flies).

The 6-day-old larvae underwent sieving with a 2 mm diameter mesh to eliminate frass, a term commonly used to describe the insect larvae feces or dejecta (28). A total of 1,800 larvae were manually extracted from the cleaned biomass and grouped into sets of 100 individuals. These groups were then weighted (0.2 ± 0.001 g—KERN, PLS, d:0.001 g) and inoculated into 18 trial boxes. The boxes were randomly assigned to one of the three manipulation treatments: hard handling (HH), soft handling (SH) and no handling (NH). Given that manipulation of the insects was at the basis of data recording, data collection methods varied among treatments. Specifically, larval survival, growth performance and bioconversion efficiency were determined for SH and HH treatments, while adult emergence and sex ratio for SH, HH and NH groups. In Figure 1, treatments and handling periods are illustrated, and their characteristics are as follows:

- HH treatment: the larvae were sampled every 4 days until the end of the larvae stage.
- SH treatment: the larvae were sampled at the end of the larval stage.
- NH treatment: larvae and prepupae were left unhandled throughout the trial.

### Ongoing sampling procedure

The following sampling procedures were conducted during the larval stage every 4 days from the beginning of the trial, specifically for the HH treatment. Each replicate was gently homogenized though manual operation and the sample was then collected in two standardized points: the upper right corner and the opposite left corner of the box. Samples were deemed representative when at least 30 larvae were counted and, if the count was lower, a second sample was collected. Larvae were extracted from the substrate using tweezers, washed with warm tap water, dried with tissue paper, and then placed into an empty box. Subsequently, individual weighing was carried out as follows: larvae were taken from the box, individually placed in the tare-box on the balance (KERN, PLS, d:0.001 g) and returned to the respective replicate. The larvae were maintained under laboratory environmental condition (20°C and 40% of relative humidity) for 30 min to facilitate the sampling operations.

### Larval stage-end identification and sampling procedures

The end of larval stage was considered when the 5% of the larvae reached the prepupal stage (~14 day old). To determine this percentage, daily inspections of the boxes from the HH and SH treatments were conducted. Once the first prepupae was identified, the sampling procedure was carried out. Specifically, both the larvae and the prepupae were extracted from the substrate using tweezers, counted, and then placed back into the box. Upon reaching the pre-determined percentage, the date was marked, and all the larvae of each replicate were extracted from the frass, counted, and used to calculate the survival rate (SR). Subsequently, larval biomass and frass were weighed (KERN, GAB-N, d:0.1 g) to calculate the growth rate (GR), the bioconversion efficiency corrected for the residue (BER), the reduction rate and waste reduction index on a fresh matter basis (21). At the end of final sampling procedures, larvae and prepupae were returned to the boxes to continue the development until the adult stage.

### Adult stage sampling operations

Since the adult stage parameters were recorded without direct manipulation of individuals, data were collected for the HH, SH and NH treatments. For each replicate, the date of the first fly emergence was recorded to calculate the development time from larva to fly (LF—time), while the emergence rate (29, 30) and the sex ratio (males-to-females) were determined on the dead flies.

### Statistical analysis

For all the recorded parameters, data were analyzed by using IBM SPSS Statistics software (V20.0.0.). Each rearing box served as a statistical unit. Data normality was evaluated with Shapiro–Wilk test, and the homogeneity of variances was evaluated using Levene’s test. All the normally-distributed data collected for the HH—SH treatments during the larval stage were subjected to independent samples t-test (BER, reduction rate and waste reduction index), while the non-parametric parameters were assessed using Mann Whitney’s U-test (development time, final average weight, total final biomass, SR, and GR). For adult parameters and for all treatments, the one-way ANOVA (*post-hoc* test: Tukey on LF—time and emergency rate) was applied in case of non-parametric data. In instances where data did not conform to a normal distribution, the non-parametric Kruskal-Wallis test was employed (sex ratio). Results were expressed as mean and standard deviation, and the level of significance considered was < 0.05.

## Results

To minimize experimental bias, the initial the weight of the 6-day-old larvae was not statistically different across treatments (*p* > 0.05; 0.0019 g ± 0.00056).

The parameters analyzed for the HH and SH treatments during the larval stage are presented in Table 1. Larvae from the HH and SH treatments exhibited no statistically significant differences, taking 8.2 days to reach 5% prepupae with an average final weight of 160 and 150 mg, respectively (*p* > 0.05). Consequently, the daily growth rate was not affected by the treatment, along with the final biomass that reached 15.1 g in the HH and 14 g in the SH groups (*p* > 0.05). No differences were observed in the SR of the larvae, which was 94.5% in the HH and 96.2% in the SH treatment (*p* > 0.05).

**Table 1:** Parameters tested for HH and SH treatments during the larval stage.

		Treatments			
Parameters	Unit	HH	SH	ST. DEV.	*p* value
Days until 5% prepupae reached	*days*	8.2	8.2	0.289	0.699
Final average weight	*mg*	160	150	0.011	0.684
Final larval biomass	*g*	15.1	14.0	1.525	0.180
SR	*%*	96.2	94.5	6.140	0.818
Growth rate	*g/d*	0.019	0.018	0.0015	0.114
BER	*%*	14.3	12.9	1.743	0.187
Reduction rate	*%*	58.2	55.9	3.276	0.581
Waste reduction index	*%/d*	7.3	7.3	0.373	0.282

SR, survival rate; GR, growth rate; BER, bioconversion efficiency corrected for residue; RR, reduction rate; WRI, waste reduction index; HH, hard handling; SH, soft handling. Results expressed on fresh matter basis.

None of the bioconversion parameters tested for the HH and SH treatments showed statistically significant differences (*p* > 0.05). In the most manipulated larvae (HH group), the BER was 14.3%, while in the SH treatment it was 12.9% (*p* > 0.05). The reduction rate of substrate in HH treatment was 58.2% and, for the SH group, it was 55.9% (*p* > 0.05). The daily waste reduction index was also statistically equal between HH and SH groups, displaying a value of 7.3% (*p* > 0.05).

Table 2 shows the results of parameters recorded during the adult stage for the NH, SH and HH treatments. The LF—time showed no statistically significant differences among treatments (*p* > 0.05), with values of 20.7 days for NH and SH groups, and of 20.8 days for the HH treatment. Similarly, the emergence rate and sex ratio also did not show any statistically significant difference among NH, SH and HH treatments (92.8%—1.1; 89.5%—1.2; 82.7%—1.2, respectively; *p* > 0.05).

**Table 2:** Parameters tested for NH, SH and HH treatments during the adult stage.

		Treatments				
Parameters	Unit	NH	SH	HH	ST.DEV.	*p* value
LF—time	*days*	20.7	20.7	20.8	0.461	0.770
Emergence rate	*%*	92.8	89.5	82.7	14.544	0.412
Sex ratio	*%*	1.1	1.2	1.2	0.337	0.854

LF—time, larva-fly time; ER, emergence rate; sex ratio, males-to-females; NH, no handling; SH, soft handling; HH, hard handling. (See all results for Table 1) Influence of three different larval handling stages on BSF life history traits.

## Discussion

To the best of the authors’ knowledge, this study represents the first attempt to evaluate the effects of three different larval manipulation procedures and their intensity on all the life stages of BSF. Currently, there is a gap in the literature regarding insect manipulation, compounded by the absence of standardized experimental protocols as suggested by Bosch et al. (21). This lack of standardization makes it challenging to compare results across different research studies. In order to assess the specific effect of manipulation procedures on the analyzed parameters, the Gainesville diet, typically used as a reference diet, was employed (21, 29–32), along with homogenous initial biomasses of 6-day-old larvae. The results of the study are discussed through comparison with scientific works designed with a similar experimental protocol and to minimize variability in findings.

The larval development time did not depend on the handling intensity (HH and SH). The 6-day-old BSF larvae subjected to both SH and HH treatments took an average of 8.2 days to reach 5% prepupae. Considering the entire development time, including the age of the larvae before the beginning of the trial, the larval stage lasted 14 days in the present study, showing a shorter duration when compared to the 17 days obtained by Miranda et al. (2), who used the Gainesville diet as substrate. The development time may be influenced by the methodology choices; specifically, Miranda et al. (2) employed a reduced feeding rate (0.7 g/larva) and manipulated the larvae every day for 9 consecutive days. Regarding feeding rates, the prolonged prepupation observed by Miranda et al. (2) could be attributed to the substantial quantity of substrate provided to the larvae (33). Nevertheless, the impact of permanent handling on the duration of the life stage may not be discounted. When the same manipulation procedures (every 4 days) are considered, the 14 days of development time align with results found in the literature (20), where brewery by-products were tested (20.1% of crude protein on dry matter). The BSF larvae require a well-balanced nutrients and appropriate protein supply to growth and become prepupae, which is around the 14–16% of crude protein on dry matter, and both the diet of the current study and of Meneguz et al. (20) contained a favorable protein percentage associated with a rapid growth (29, 34).

The handling intensity did not influence the final average larval weight (HH, 160 mg; SH, 150 mg), aligning with the average weights recorded in other studies conducted on the same diet (from 150 to 160 mg, on fresh matter basis) (2, 35, 36).

Nguyen et al. (16) assessed the effects of the manipulation on BSF larvae fed various organic waste, including pork liver with a protein content of 19 and 3% of ether extract on dry matter, which is a diet most comparable to the Gainesville diet. As concern the manipulation, the feed from the previous day was removed and replaced with fresh feed, and all the larvae were counted to record SR, while weight and length were measured at least on 30 larvae per replicate (16). In contrast, “unhandled” larvae were never counted, weighed or measured. Furthermore, the feed was consistently replenished without removal for the unhandled treatment (16). According to this experimental protocol, the authors observed a higher final larval weight in the unhandled larvae compared to the handled ones (about 160 mg and 90 mg, respectively) (16). However, the observed differences in average larval weight between the handled and unhandled larvae by Nguyen et al. (16) may be attributed to the chosen methodology rather than to the manipulation itself. In particular, by adding a new feed daily to both treatments and removing the residual feed only from the manipulated group, the unmanipulated larvae may have ingested a larger amount of substrate, consequently displaying a higher final weight.

Regarding larval biomass, this parameter is the product of high SR and adequate average larval weight. The SR could be considered a coarse proxy indicator of individual health and welfare status (5). On the other side, since there are not any certified welfare markers, it may be used as parameter to evaluate the suitability of the management condition. In literature, recent reviews (37, 38) that compared different studies conducted with BSF have reported SRs higher than 80%. If the Gainesville diet is considered, the results obtained in the current study are comparable to the SR of Arabzadeh et al. (32), who performed the sampling operations every two days.

As concern the GR, the results of the present study are higher when compared to the one from Pliantiangtam et al. (35) (0.018 and 0.015 g/d, respectively; on fresh matter basis), which used the Gainesville diet as rearing substrate. Although the feeding rate is not indicated, the lowest GR observed by Pliantiangtam et al. (35) may be related to the experimental strategies chosen during the trial setup. In fact, in the current study, the end of the larval stage was considered when 5% of prepupae appeared, while Pliantiangtam et al. (35) opted for 40%. It is known that in the last instar of growth, the larvae stop feeding and lose weight (39), and since the GR is calculated as a difference between the average final and initial larval weight, the presence of the largest amount of prepupae lowered the final values, thus having a direct effect on the considered parameter. Moreover, since the 40% of prepupae was selected as cut-off of the larval stage, the development time lasted 1 day more, thus further reducing the value (35).

At the end of the experiment, to calculate the real conversion efficiency of BSF larvae—that considers not only the diet consumed but also the exuviae and excreta produced—a BER formula was employed, as suggested by Bosch et al. (21). In the literature, there are several formulas used to express conversion efficiency as the feed conversion ratio (FCR) or the efficiency of conversion of digested or ingested food (ECD—ECI). However, such formulas could lead to an overestimation if the fraction of unconsumed feed (FCR, ECI) or only if the final biomass (ECD) is considered (21, 34). The BER shows similarities (HH, 14.3%; SH, 12.9%, fresh matter basis) with what was reported by Arabzadeh et al. (32) (14.3%, fresh matter basis). Therefore, it is possible to exclude the effect of handling and attribute the observed differences to the lower feeding rate adopted by Arabzadeh et al. (32).

Strictly related to the BER, the waste reduction index and reduction rate were calculated and, also in this case, no statistically significant differences related to the handling procedures were found. Starting with the reduction rate, the review by Surendra et al. (40) described several studies with a minimum RR of 25% for human feces and maximum RR of 72% (on dry matter %) with soybean curd residues used as growing substrates. The Gainesville diets employed as rearing substrate by Arabzadeh et al. (32) and in the current study showed a reduction rate (both on fresh matter basis) of 70.5 and 57.1% (as mean of HH and SH treatments), respectively. This huge diversity in terms of reduction rate could be due to the different feeding rates (33), the larger number of larvae reared (41), and from the 40% cut-off that allowed more time and opportunity for the remained 5^th^ instar larvae to reduce the substrate.

High values of waste reduction index are indicative of a good reduction of the substrate due to larval efficiency (42). The larval ability to reduce substrate depends on different aspects, such as the nutritional composition of the diet, the feeding rate, the time spent by the larvae on the substrate and, obviously, the environmental conditions. Considering the diet used as rearing substrate, the results obtained by Pliantiangtam et al. (35) are the better values to compare with the current study, albeit the used methodologies were different. Despite Pliantiangtam et al. (35) having not sampled the larvae throughout the trial, the waste reduction index was the lowest (4.9 g/d, on fresh matter basis), and, for this reason, it is possible to rule out the manipulation as the main cause. Even if the sampling procedures are the same, the nutrient composition of the diet may influence the waste reduction index. This hypothesis could be confirmed when the results of the present study are compared to the ones of Meneguz et al. (20), which used the same manipulation of HH treatment. Specifically, the larvae fed on brewers spent grain showed a lower the waste reduction index (5.3 g/d, on fresh matter basis) than the HH group (7.3 g/d). This outcome may be probably linked to the high percentages of crude fiber of beer by-product, which has been reported to slow down the digestibility of the substrate by BSF larvae (43, 44).

Finally, noteworthy is the trend of numerically higher values of HH treatment than SH for final larval biomass weight, SR, BER and reduction rate parameters. However, one possible explanation may be related to the methodological choice made during the sampling operations. In fact, while SH treatment was manipulated only with the achievement of 5% prepupae (14-day-old larvae), the HH replicates were manipulated every 4 days until 5% prepupae were reached. Specifically, during the sampling operations before collecting the larvae, the additional homogenization procedures have reduced the firmness of the substrate by increasing the available surface area, likely promoting the growth of beneficial microbes and nutrient availability and, consequently, the larval growth and bioconversion capacity (42, 45).

The parameters recorded and calculated during the adult stage were evaluated also for the third treatment—the NH. No differences in term of LF—time, emergency rate and sex ratio were observed among the treatments. Considering that the NH treatment was never manipulated, it is possible to exclude that the handling procedures affected these parameters. In a previous study conducted by Bellezza Oddon et al. (29), which match almost completely to the present study in terms of substrate and rearing condition, the LF—time was 3 days higher than the recorded times (NH—SH, 20.7/d; HH, 20.8/d). The sampling operations used were the same as those performed in Bellezza Oddon et al. (29), so the effect of handling can be ruled out as a cause of the observed differences. However, the developmental and productive performance of BSF may be affected by several factors, including quality and quantity of feed or larval density (46). Given the similar and favorable macronutrient composition of the Gainesville diets, the quality of the substrate may also be excluded as the main cause. Moreover, the effect of larval density could also be excluded, as up to 5 larvae/cm^2^ would not significantly affect larval development time and weights (47). Similarly, the effect of photoperiod may also rule out as compared to the difference in LF—time observed with the study of Bellezza Oddon et al. (29). Specifically, the same photoperiod employed in the present work (0:24—light:dark) than (16:8—light:dark) by Bellezza Oddon et al. (29) required the longest times (expressed as accumulated degree hours—ADH) to complete egg-to-adult development when comparing with photoperiods of 8:16 and 12:12 light:dark (48). Differently, among the possible causes that could explain the observed LF—time difference, is the lower feeding rate employed during the larval stage in Bellezza Oddon et al. (29), which could possibly delay the BSF larvae development (46) and, consequently, the emergence of the flies.

Moving on with the emergency rate, treatments showed 92.8% of flies emerged for NH, 89.5 and 82.7% for SH and HH treatments, respectively. Despite the absence of statistical differences, it is worth noting that the numerical trend displayed a reduction in emergence of BSF flies as manipulations increased. Such a numerical difference could be related to the different temperature the replicates were exposed to during the sampling operations. The temperature may have a significant impact on the development of the black soldier fly. Already in 1998, Polak (49) predicted that the effect of abiotic stressors (e.g., temperature) could be able to modify the physiological state of organisms, leading them to changes in welfare and lethality. Along these lines, Holmes et al. (50) showed that at 19°C the overall percentage of successfully emerged adults was 31.9% only. Similarly, it is possible to assume that the shift from a climate chamber (T: 28 ± 0.5°C; RH: 70 ± 5%) to a laboratory condition, with standard environmental temperatures of 20°C (30 min) repeated over time, may be inappropriate for the development of a tropical, temperate-warm region fly (51).

The analysis of the relationship between the sex (males and females) was aimed at investigating a possible sensitivity of sex to manipulation procedures. According to Ernsting & Isaaks (52), the evolution of sexual dimorphism in the animal kingdom is governed by multiple types of selection operating simultaneously, leading, for example, to competition between members of the same or opposite sex for limited resources, preference for some members of the opposite sex, and selection on the basis of differing reproductive roles. No statistically significant difference between treatments were detected, with a prevalence of male flies in line with studies by Holmes et al. (53) and Bellezza Oddon et al. (30), thus refuting the initial hypothesis.

## Conclusion

Handling of larvae during BSF larvae feeding experiments could affect the life history traits. Independently of application scale (small or large), most often there are mandatory essential procedures that require larval handling. Despite the large number of insects reared and given the expected global development of BSF breading facilities, there are few regulations related to insect rearing welfare. Overall, the present results suggest that larval handling does not have an influence on the growth and development of BSF larvae. However, further studies are needed to confirm the results herein obtained and clarify whether the numerically trends shown for both larval and adult stage performance parameters can be refuted or confirmed by varying genetics, application scale, or implemented manipulation procedures.

## Data availability statement

The raw data supporting the conclusions of this article will be made available by the authors, without undue reservation.

## Ethics statement

The manuscript presents research on animals that do not require ethical approval for their study.

## Author contributions

ZL: Data curation, Formal analysis, Investigation, Resources, Writing – original draft. LG: Conceptualization, Investigation, Methodology, Writing – review & editing. IB: Conceptualization, Investigation, Methodology, Supervision, Writing – review & editing. AR: Investigation, Resources, Writing – review & editing. SB: Conceptualization, Data curation, Formal analysis, Investigation, Methodology, Project administration, Resources, Supervision, Writing – original draft, Writing – review & editing.
